# Evidence of recombination of vaccine strains of lumpy skin disease virus with field strains, causing disease

**DOI:** 10.1371/journal.pone.0232584

**Published:** 2020-05-13

**Authors:** Alexander Sprygin, Yana Pestova, Olga Bjadovskaya, Pavel Prutnikov, Nikolay Zinyakov, Svetlana Kononova, Olga Ruchnova, Dmitiy Lozovoy, Ilya Chvala, Aleksandr Kononov

**Affiliations:** Federal Center for Animal Health, Vladimir, Russia; Bangladesh Agricultural University, BANGLADESH

## Abstract

Vaccination against lumpy skin disease (LSD) is crucial for maintaining the health of animals and the economic sustainability of farming. Either homologous vaccines consisting of live attenuated LSD virus (LSDV) or heterologous vaccines consisting of live attenuated sheeppox or goatpox virus (SPPV/GPPV) can be used for control of LSDV. Although SPPV/GTPV-based vaccines exhibit slightly lower efficacy than live attenuated LSDV vaccines, they do not cause vaccine-induced viremia, fever, and clinical symptoms of the disease following vaccination, caused by the replication capacity of live attenuated LSDVs. Recombination of capripoxviruses in the field was a long-standing hypothesis until a naturally occurring recombinant LSDV vaccine isolate was detected in Russia, where the sheeppox vaccine alone is used. This occurred after the initiation of vaccination campaigns using LSDV vaccines in the neighboring countries in 2017, when the first cases of presumed vaccine-like isolate circulation were documented with concurrent detection of a recombinant vaccine isolate in the field. The follow-up findings presented herein show that during the period from 2015 to 2018, the molecular epidemiology of LSDV in Russia split into two independent waves. The 2015–2016 epidemic was attributable to the field isolate. Whereas the 2017 epidemic and, in particular, the 2018 epidemic represented novel disease importations that were not genetically linked to the 2015–2016 field-type incursions. This demonstrated a new emergence rather than the continuation of the field-type epidemic. Since recombinant vaccine-like LSDV isolates appear to have entrenched across the country’s border, the policy of using certain live vaccines requires revision in the context of the biosafety threat it presents.

## Introduction

Lumpy skin disease virus (LSDV) is a capripoxvirus belonging to the family *Poxviridae*. It comprises a viral genome constituting double-stranded DNA of ~150 kbp [1]. Moreover, LSDV shares over 97% sequence identity with viruses of the genus *Capripoxvirus*, including the sheep and goat pox viruses. Previous studies examining the genetic architecture have demonstrated that capripoxviruses and other members of the family *Poxviridae* might have originated from a single ancestral strain through gene gain and loss [2–5].

Cattle and water buffalos are susceptible to LSDV virus and may develop pox lesions on the skin and internal organs when infected [6,7]. Outbreaks are accompanied by high morbidity but low mortality, inflicting considerable economic losses [8,9].

The disease can spread in two ways via arthropod-borne transmission and movement of infected animals [10]. Spread through contact transmission and fomites are considered ineffective but remain a possible route of transmission [11,12]

LSDV was considered to be restricted to Southern Africa; however, by 1956, it spread to Central and Eastern Africa and, by 1989, to the Middle East [13,14]. Since 2015, LSD outbreaks have spread into new territories in the Northern hemisphere such as Serbia and Russia, among other countries [15,16].

Recent incursions into the Balkans, Europe, and Russia have necessitated the rapid implementation of vaccination campaigns aiming at controlling disease outbreaks and further spread. Vaccination is the only effective control strategy and remains the main approach to maintaining animal health and preventing economic loss. However, there exist multiple gaps in the understanding of the epidemiology, genetic features, and transmission mechanisms of LSDV, which significantly impede the development of control strategies [7]. Moreover, the spread of LSDV into new climate zones requires further research to evaluate its transmission and prevalence in new environmental contexts. Although the LSDV isolates recovered in the Balkans, Europe, and Russia appear to be of monophyletic origin [17–19], the disease manifestation tends to vary depending on geographical location and breed characteristics [20]. Moreover, the genetic background underlying the observed phenotype remains poorly understood.

Vaccines against LSDV are available as live attenuated LSDV isolates (e.g., the Neethling and KSGP vaccines) and sheep /goat pox virus (SPPV/GPPV)-based preparations [7]. The choice of vaccine is complicated by conflicting findings of their safety, efficacy, and transmission capacity [21–23]. Considering the replication competence of live vaccines [24], the risk of coinfection and subsequent recombination are considered to be low for these poxvirus vaccines and made it the preferred, choice in most countries. For instance, the EU and Balkans opted for the attenuated Neethling vaccines, whereas Turkey, Georgia, and Russia resorted to SPPV vaccines that establish cross-protection against LSDV [25].

A total of 436 outbreaks were reported by the Russian Federation to the World Organization for Animal Health: 17 in 2015, 313 in 2019, 42 in 2017, and 64 in 2018 [26]. However, the LSDV scenario in Russia, where only SPPV vaccines are used, has become unexpectedly complex in light of the recent evidence regarding the occurrence of multiple vaccine-like strain incursions since 2017 [27]. In Russia, vaccination against LSD started as a ring vaccination in 2016 in affected zones, and it has been remaining in place in previously and newly affected zones. Typically, LSD-free but at-risk regions can opt out of vaccination to act as surveillance zones. However, once the disease occurs, the region-wide vaccination is mandatory. In 2018, all regions that had been affected in 2015–2017 were previously vaccinated.

Importantly, despite the use of an SPPV-based vaccine, vaccine-related diseases were first reported in 2017, in regions of a geographically distinct area bordering Kazakhstan practicing live attenuated LSDV vaccination [28]. In the same year, a naturally occurring vaccine recombinant isolate—Saratov/2017—was first isolated from the same geographic region [29]. This naturally occurring hybrid predominantly comprised the Neethling vaccine genome with interspersed fragments of a field-type virus with signatures resembling those of a KSGP-like variant. Moreover, a much wider spectrum of vaccine-like isolates was reported in Saratovskaya Oblast in the same 2017 year, close to the border of Kazakhstan, from where recombinant Saratov/2017 was recovered [30].

The present study delineated the genetic diversity of LSDV strains recovered in Russia during 2015–2018 based on the *RPO30* and *GPCR* targets, which were recently shown to be reliable loci for detecting recombination [29]. In the absence of live attenuated LSDV vaccines in countries neighboring Russia in 2016, the Russian LSD outbreaks were solely attributed to LSDV field isolates. After the introduction of such vaccines in Kazakhstan in 2017, vaccine-like isolates underwent multiple dispersal events across a wide area toward the East along the Russian border, resulting in a new epidemiological wave of genetically different LSDV.

## Materials and methods

### Ethics statement

This study did not involve any handling of live animals; thus, no approval was required from the ethics committee of the Federal Center for Animal Health (Vladimir, Russia).

### Samples and PCR

To obtain the 21 LSDV isolates from different regions, blood samples were collected between 2015–2018 from cows presenting clinical signs of LSDV. One sample per region was used if the identical virus was found among multiple isolates within a region. Blood samples were collected from animals presenting clinical signs of LSDV characterized by abundant nasal discharge, fever, and skin lesions measuring 1.2–3.8 cm in diameter, frequently coalescing together on the skin. Total DNA extraction was performed using the DNeasy Blood & Tissue Kit (Qiagen, Germany), as per the manufacturer’s instructions. The final elution was conducted with nuclease-free water. The presence of LSDV DNA was initially confirmed by screening with PCR reported previously [31]. The isolates used in the study are outlined in Table 1.

**Table 1:** LSDV isolates sequenced in this study.

Isolate/year of identification	Region of Russia	GenBank accession number	
		*RPO30*	*GPCR*
Chechnya/2015	Chechnya	MK765509	MK765530
Volgograd/2016	Volgograd	MK765510	MK765531
Voronezh/2016	Voronezh	MK765511	MK765532
Krasnodar/2016	Krasnodar	MK765512	MK765533
Stavropol/2016	Stavropol	MK765513	MK765534
Chechnya/2016	Chechnya	MK765514	MK765535
Ingushetiya/2016	Ingushetiya	MK765515	MK765536
Karachaevo-Cherkessiya/2016	Karachaevo-Cherkessiya	MK765516	MK765537
Kabardino-Balkariya/2016	Kabardino-Balkariya	MK765517	MK765538
Kalmykiya/2016	Kalmykiya	MK765518	MK765539
Tambov/2016	Tambov	MK765519	MK765540
Rostov/2016	Rostov	MK765520	MK765541
Ryazan/2016	Ryazan	MK765521	MK765542
Abkhazia/2016	Abkhazia^1^	MK765522	MK765543
Kazakhstan/2016	Kazakhstan^2^	MK765523	MK765544
Omsk/2018	Omsk	MK765524	MK765545
Omsk2/2018	Omsk	MK765525	MK765546
Sverdlovsk/2018	Sverdlovsk	MK765526	MK765547
Samara/1461/2018	Samara	MK765527	MK765548
Samara/1462/2018	Samara	MK765529	MK765550
Chelyabinsk/2018	Chelyabinsk	MK765528	MK765549

^1^ A neighboring country in Caucasus affected in 2016

^2^ A neighboring country in Central Asia affected in 2016

### Phylogenetic analysis

Nucleotide sequencing was performed at the *RPO30* and *GPCR* genes of LSDV from real-time PCR positive LSDV samples. For sequencing, PCR products were amplified as previously described [29], followed by purification using QIAquick Gel columns (Qiagen, Germany). Sequencing was performed using the amplification primers using an automated sequencer (ABI Prism 3130 Genetic Analyzer; Applied Biosystems, USA). Each sample was sequenced at least 20 times to ensure authenticity. The resulting representative sequences were used in the phylogenetic analysis.

Nucleotide sequences of the RPO30 gene of LSDV strains were downloaded from the NCBI database (www.ncbi.nlm.nih.gov). Alignments of the selected sequences were performed using the ClustalW algorithm in BioEdit Sequence Alignment Editor 7.0.5.3 [32] using default parameters. Following alignment, the sequences were manually truncated to obtain common start and end positions. Phylogenetic analyses were performed using MEGA 6.06 (http://www.megasoftware.net) using the neighbor-joining approach [33] with 1,000 bootstrap replicates. Gaps were treated as complete deletions. Only nodes with bootstrap values >70% are presented. Consensus sequences were assembled using the Staden Package [34].

### Epidemiological analysis

Following the genetic sequencing of isolates obtained from 2015–2018, a series of maps was created to track the progress of LSDV epidemiology over time. Additionally, a simulation involving the emergence of the variants over time was constructed using QGIS 3.4.12 'Madeira. [35] and the free online software Icecream Screen Recorder (https://icecreamapps.com). Finally, the association between the frequency of outbreaks and month of occurrence were analyzed.

## Results

### Nucleotide phylogenetic analysis

The *RPO30* and *GPCR* genes of the 21 LSDV isolates were sequenced. The accession numbers of the corresponding sequences are listed in Table 1.

**Fig 1:**
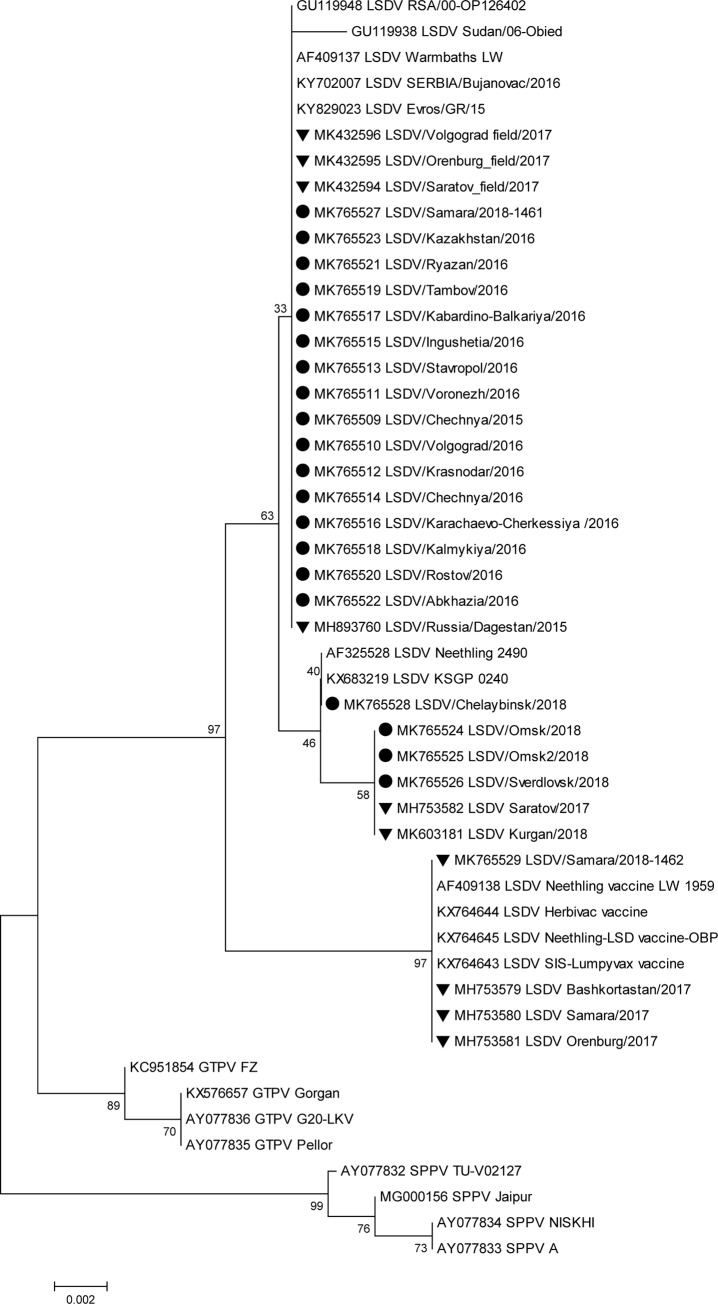
Unrooted phylogenetic tree showing the relationships between the identified LSDVs and currently available capripoxvirus nucleotide sequences of the *RPO30* locus in the GenBank database. Isolates sequenced in this study are indicated with a circle, and the Russian isolates present in GenBank (not sequenced herein) are indicated with a triangle. (LSDV: Lumpy skin disease virus, SPPV: Sheeppox virus, GTPV: Goatpox virus).

**Fig 2:**
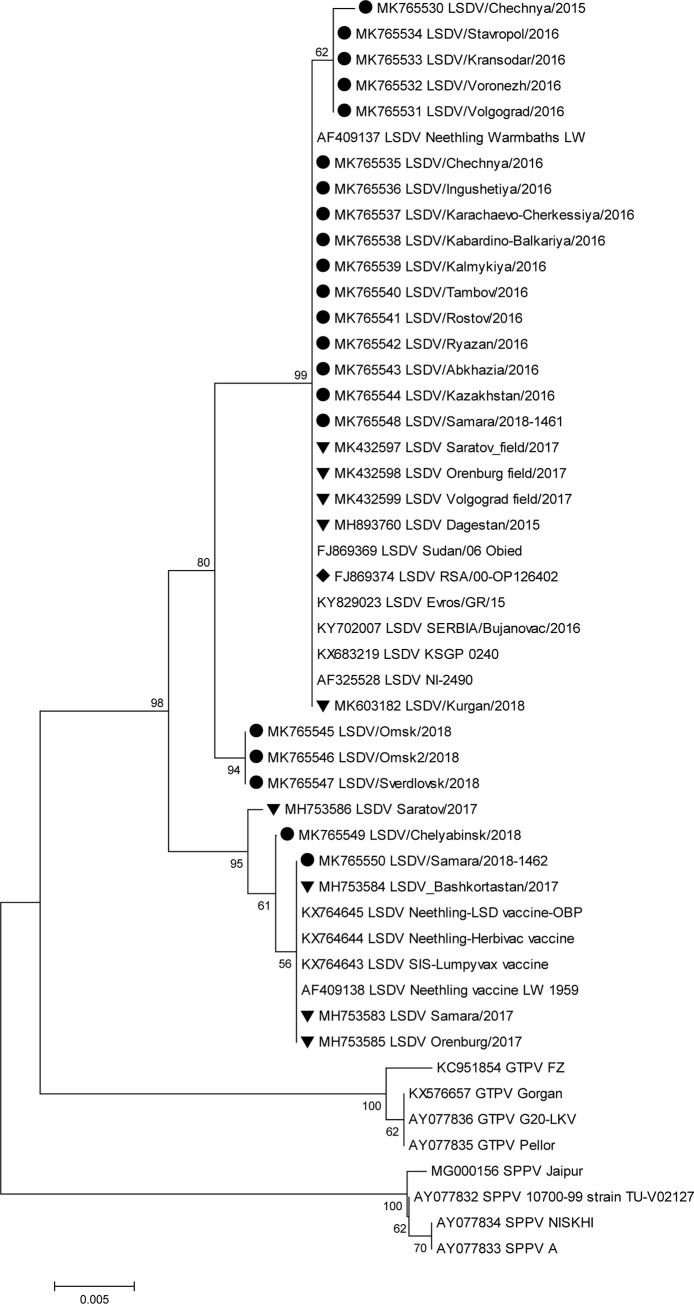
Unrooted phylogenetic tree showing the relationships between the identified LSDVs and currently available capripoxvirus nucleotide sequences of the *GPCR* locus in the GenBank database. Isolates sequenced in this study are indicated with a circle, and the Russian isolates present in GenBank (not sequenced herein) are indicated with a triangle. (LSDV: Lumpy skin disease virus, SPPV: Sheeppox virus, GTPV: Goatpox virus).

Isolates obtained in 2015 and 2016 shared 100% identity based on the *RPO30* locus and were clustered into one field isolate group (Fig 1). In contrast, the *GPCR* locus of the same isolates was variable. The isolates Chechnya/2015, Stavropol/2016, Krasnodar/2016, Voronezh/2016, and Volgograd/2016 were grouped in different sublineages, sharing 99.7%–99.8% similarity with the field isolate Dagestan/2015, (Fig 2). The *GCPR* locus among this sublineage showed 99.8%–100% similarity (Fig 2). LSDV isolates from 2018 showed separate clustering patterns for both loci (Figs 1 and 2).

Both loci (*RPO30* and *GCPR*) of the Russian field isolate Samara/1462/2018 were identical to the vaccine isolate in the commercial product Lumpyvax (Figs 1 and 2). Sequence analysis of the *RPO30* loci indicated that the Russian field isolate Samara/1461/2018 shared 99.5% and 99.1% similarity with the Russian field isolate Dagestan/2015 (isolate not sequenced herein) [18] and KSGP-like Kurgan/2018 isolate (isolate not sequenced herein) [36], respectively. The *GCPR* locus of the Samara/1461/2018 isolate contained the CTCAGTACAATT insertion site and showed 98.4% and 100% similarity with Dagestan/2015 (isolate not sequenced herein) and KSGP-like Kurgan/2018 (isolate not sequenced herein), isolates respectively.

The recent Russian field isolates Omsk/2018 (both), Sverdlovsk/2018, and Chelyabinsk/2018 displayed inconsistent agreement across the two loci, although Chelyabinsk/2018 closely matched the recombinant Russian isolate Saratov/2017 (isolate not sequenced herein), with 99.6% and 99.3% similarity for *GPCR* and *RPO30* loci, respectively. There was the same level of similarity between the isolates Chelyabinsk/2018 and Kurgan/2018 (isolate not sequenced herein) for *RPO30*. Based on the *RPO30* locus, the isolates Omsk/2018, Omsk2/2018, and Sverdlovsk/2018 were grouped with the recombinant isolates Saratov/2017 and KSGP-like Kurgan/2018 (isolate not sequenced herein). The similarity level among Omsk/2018, Omsk2/2018, and Sverdlovsk/2018 was 99.5%–99.7% and that between Saratov/2017 and Kurgan/2018 was 99.3%–99.7%. The isolates-Omsk/2018, Omsk2/2018, and Sverdlovsk/2018 retained the CTCAGTACAATT indel at the *GPCR* locus, which is typical of the vaccine and KSGP-like isolates—did not align with either the field or the vaccine groups (Fig 2), and formed a subcluster related to the field group, with 80% bootstrap support. Moreover, Sverdlovsk/2018 shared 99.2% and 97.7% identity with the recombinant isolate Saratov/2017 (isolate not sequenced herein) and Dagestan/2015 (isolate not sequenced herein), respectively.

### Amino acid phylogenetic analysis

**Fig 3:**
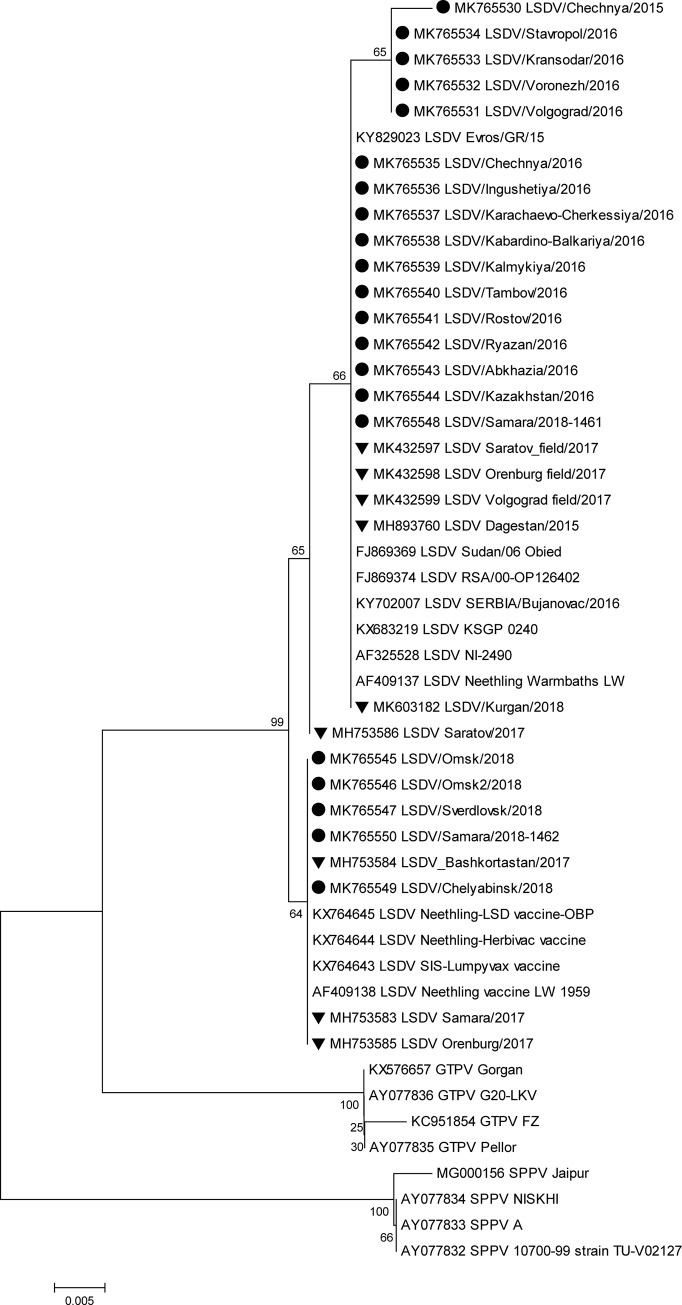
Unrooted phylogenetic tree showing the relationships between the identified LSDVs and currently available capripoxvirus amino acid sequences of *GPCR* in the GenBank database. Isolates sequenced in this study are indicated with a circle, and the Russian isolates present in GenBank (not sequenced herein) are indicated with a triangle. (LSDV: Lumpy skin disease virus, SPPV: Sheeppox virus, GTPV: Goatpox virus).

Based on the *GPCR* target, the Russian field isolates in 2016 were all identical at the amino acid level and formed a large group comprising the current field isolates detected worldwide (Fig 3). The isolates Chechnya/2015, Stavropol/2016, Krasnodar/2016, Voronezh/2016, and Volgograd/2016 were clustered with 0.04% difference from the major group, whereas Chechnya/2016 showed 0.06% difference from the major group.

The recent Russian isolates Omsk/2018 (both), Samara1462/2018, Sverdlovsk/2018, and Chelyabinsk/2018 were clustered in the vaccine group with 100% similarity. Samara1461/2018 was identical to Kurgan/2018 (isolate not sequenced herein) [36] and the KSGP vaccine.

**Fig 4:**
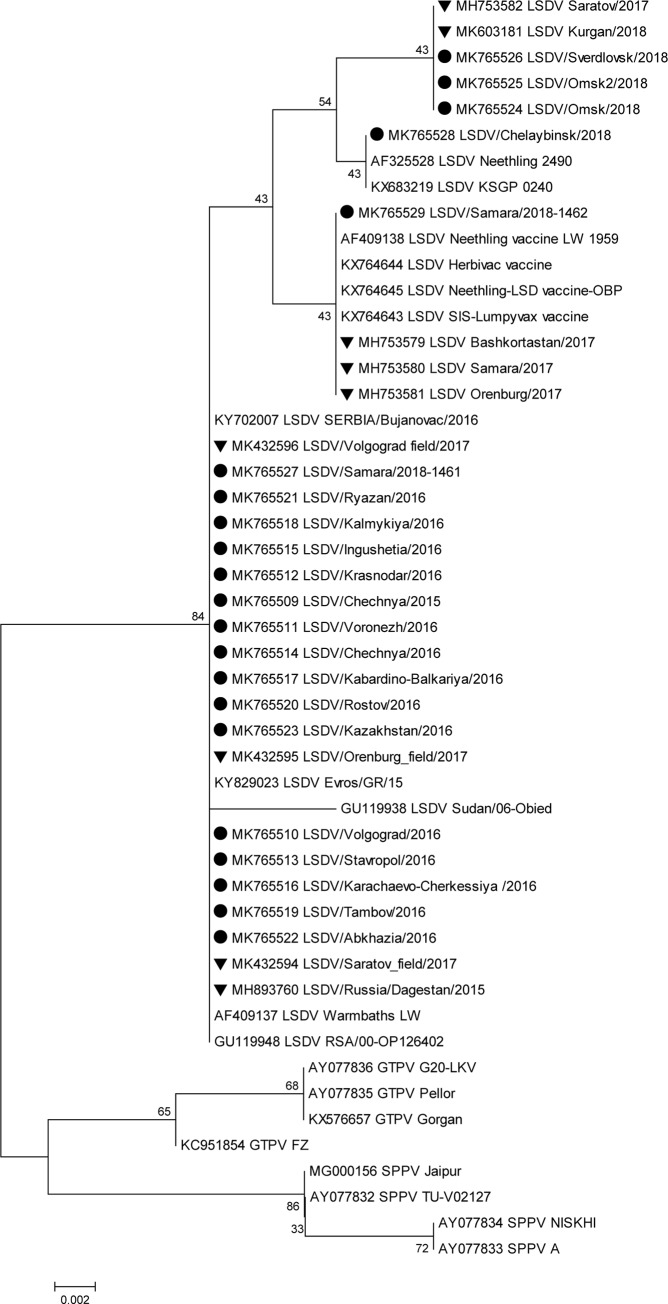
Unrooted phylogenetic tree showing the relationships between the identified LSDVs and currently available capripoxvirus amino acid sequences of *RPO30* in the GenBank database. Isolates sequenced in this study are indicated with a circle, and the Russian isolates present in GenBank (not sequenced herein) are indicated with a triangle (LSDV: Lumpy skin disease virus, SPPV: Sheeppox virus, GTPV: Goatpox virus).

Based on *RPO30* gene analysis, isolates from 2016 and Samara/1461/2018 showed 100% homology at the amino acid level and were grouped with the field isolate sequences obtained from GenBank (Fig 4).

Samara/1462/2018 fell into the vaccine group with 100% identity. Sverdlovsk/2018 and Omsk/2018 (both) were grouped with KSGP-like Kurgan/2018 and recombinant Saratov/2017 (isolate not sequenced herein) isolates with 100% homology, and Chelyabinsk/2018 was situated closer to the KSGP vaccine with 100% identity.

### LSD epidemiology during 2015–2018

**Fig 5:**
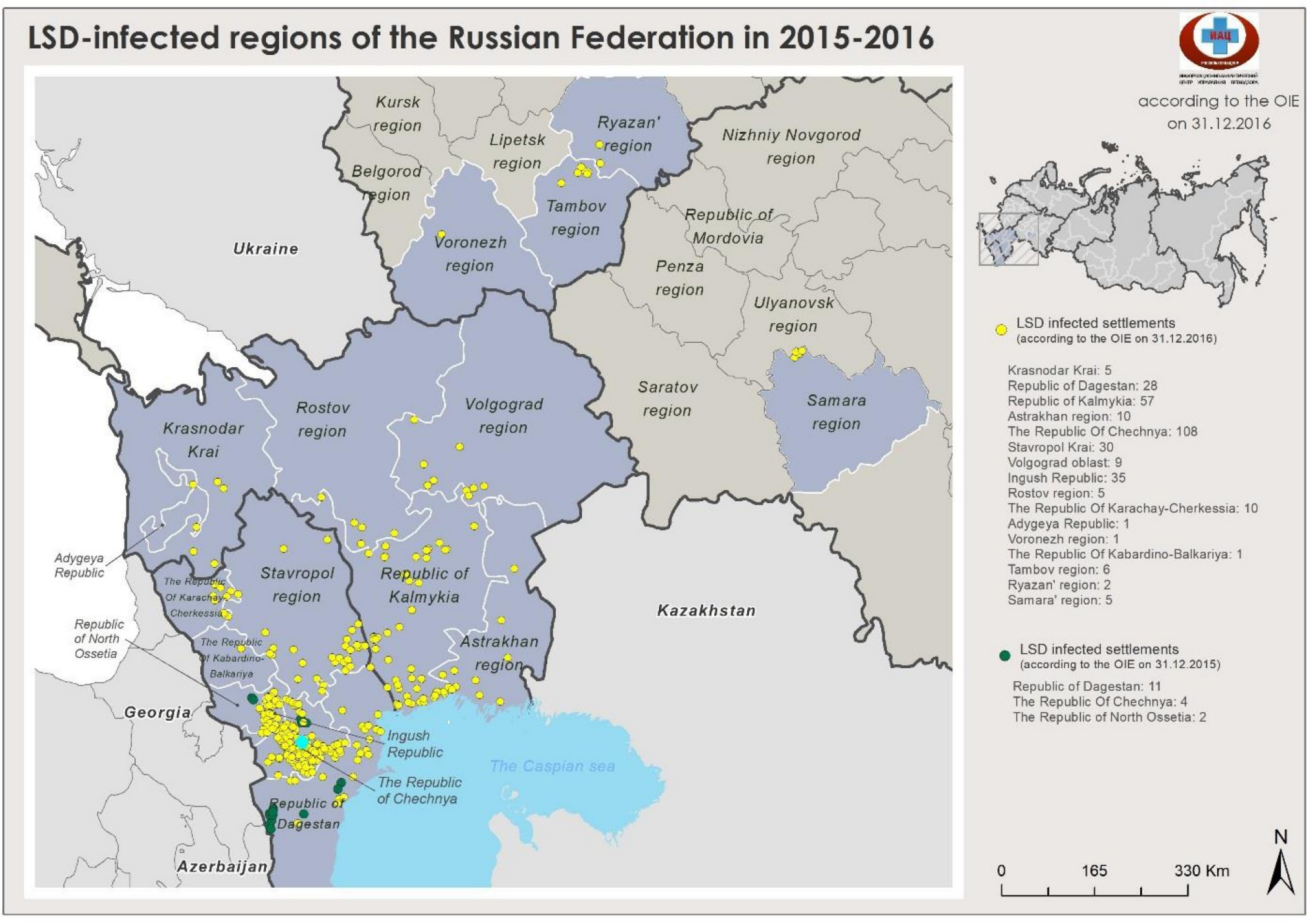
Map of LSD outbreaks during 2015–2016 (LSD: Lumpy Skin Disease).

**Fig 6:**
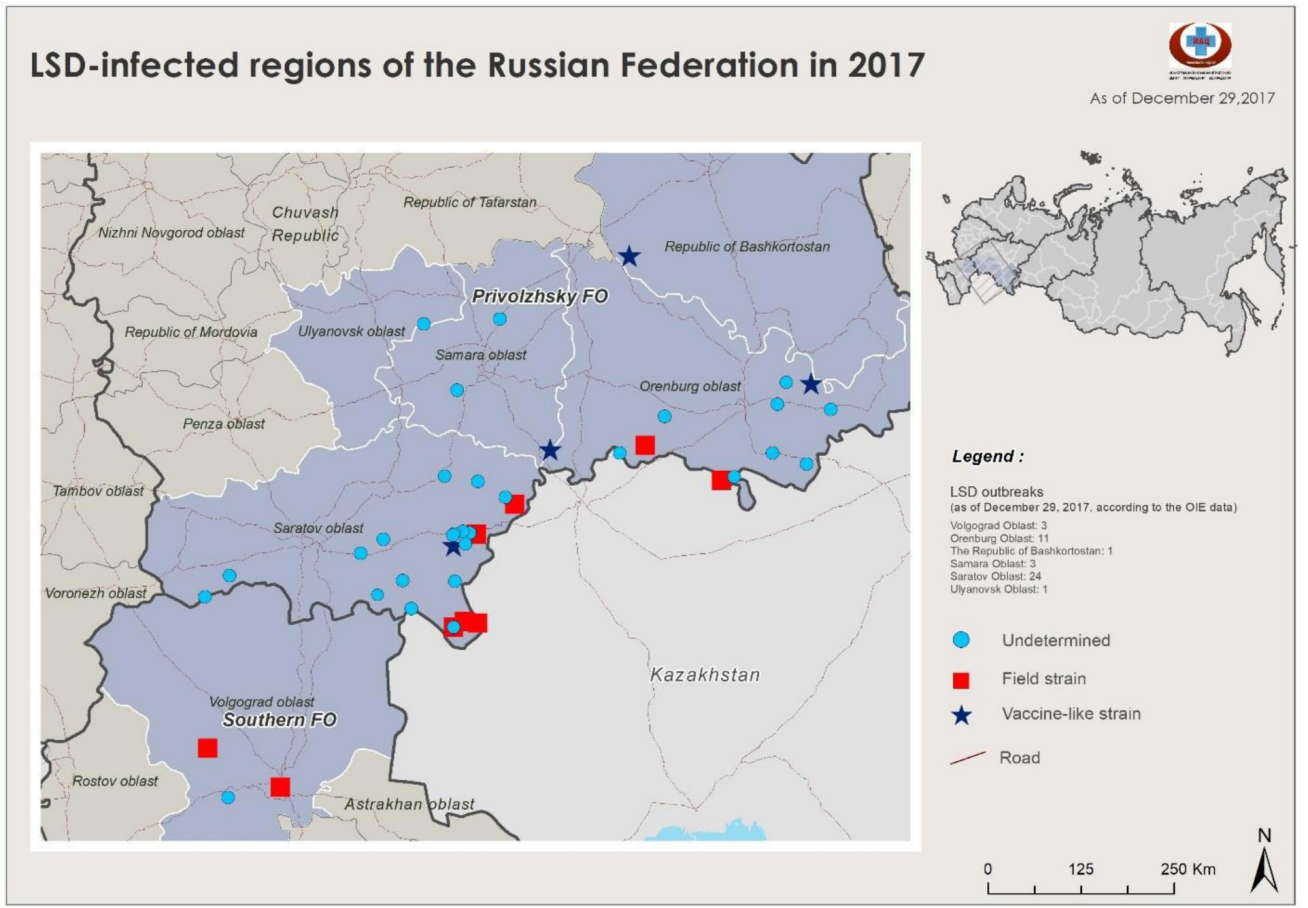
Map of LSD outbreaks in 2017 (LSD: Lumpy Skin Disease).

**Fig 7:**
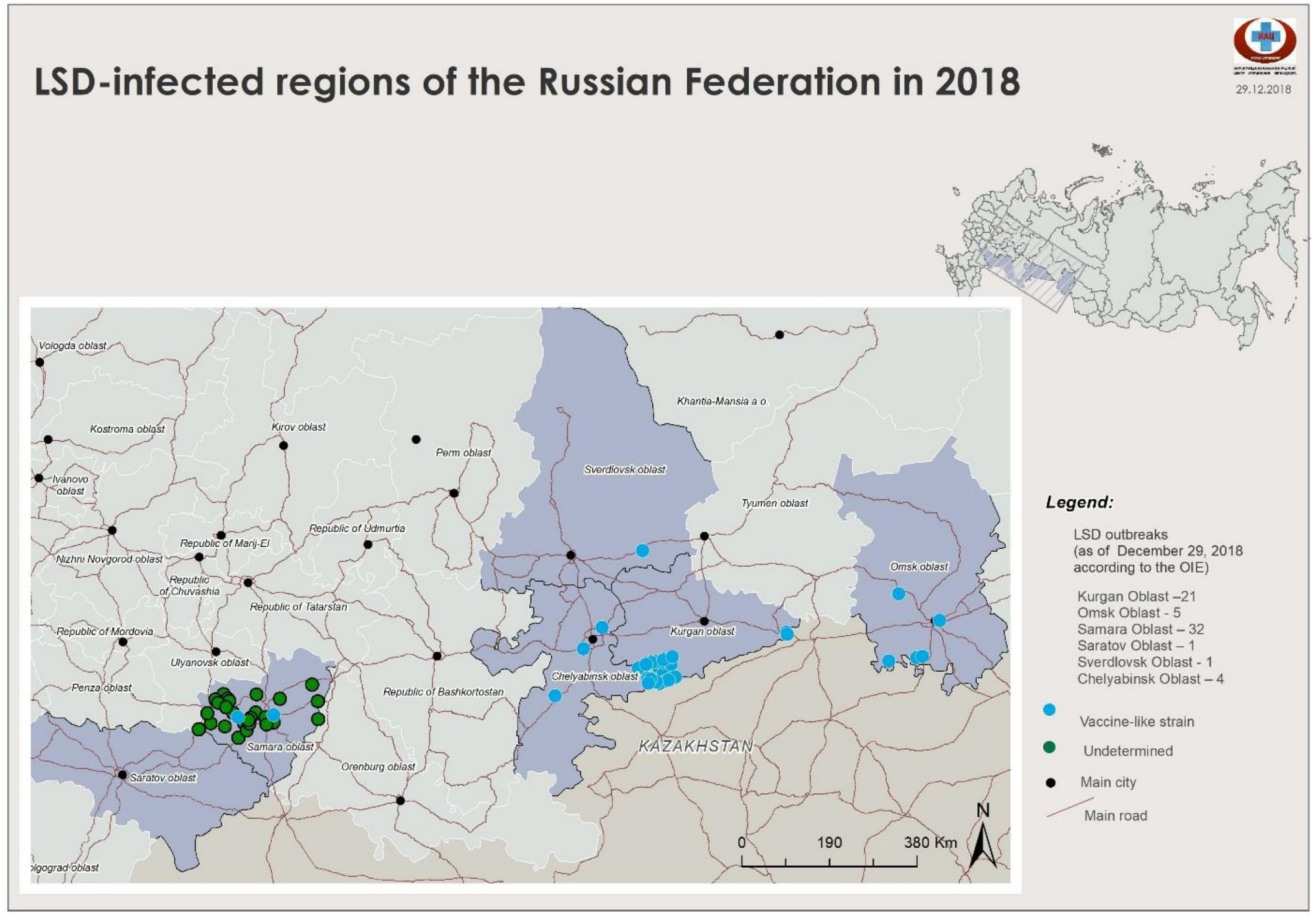
Map of LSD outbreaks in 2018 (LSD: Lumpy Skin Disease).

**Fig 8:**
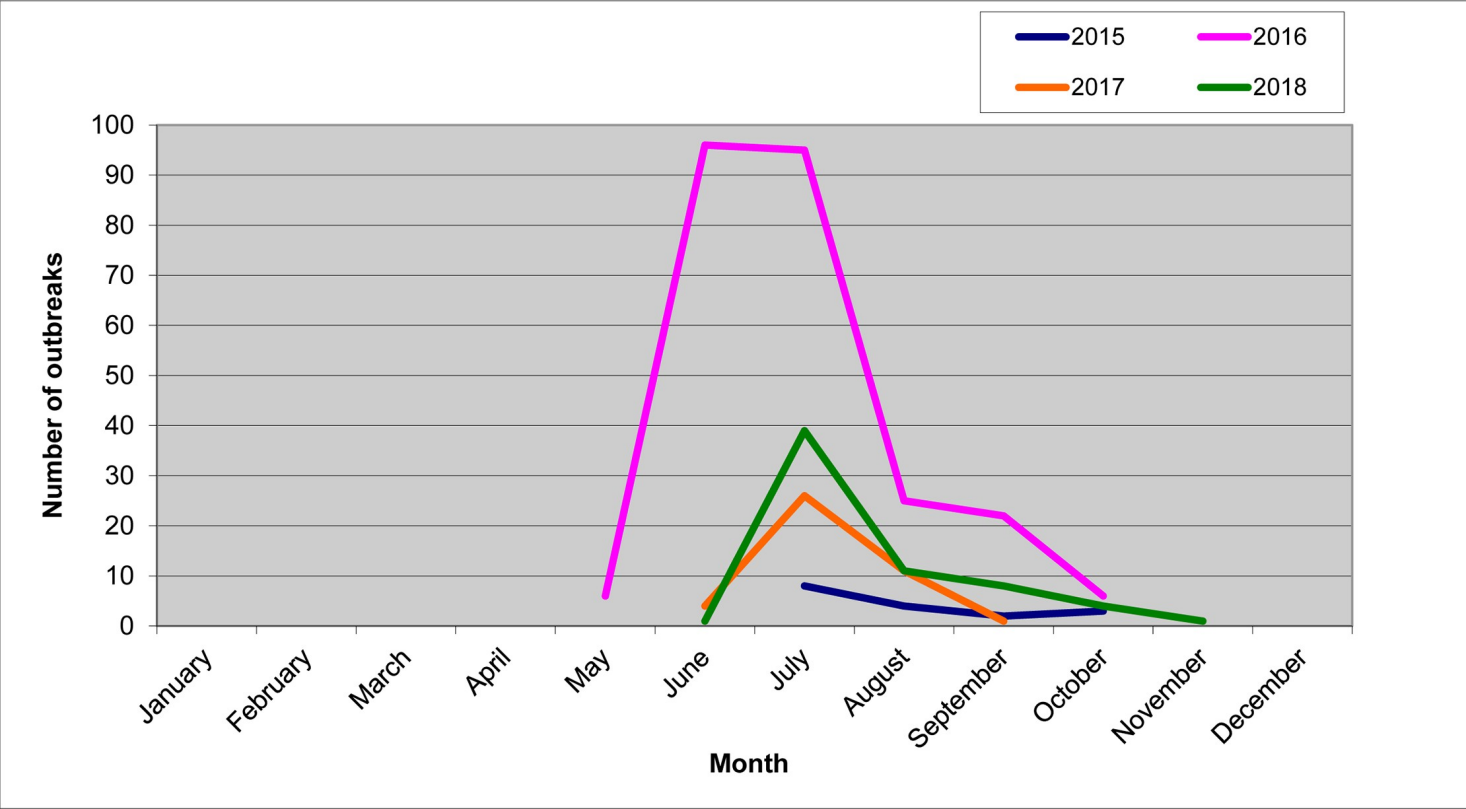
Seasonality of LSD outbreaks during 2015–2018.

The occurrence of LSDVs of differing genotypic origins over time are shown in Figs 5–7. Two outbreaks were prominent: the 2015–2016 period (Fig 5) and the 2017–2018 period (Figs 6 and 7). The outbreaks in 2015/2016 was mainly caused by field isolate while the 2017 outbreak represents a transient period with a mix of circulating variants (Fig 6); and the 2018 outbreak was caused by recombinant vaccine-like isolates (Fig 8). No outbreak caused by a field isolate was identified in 2018.

The LSDV outbreaks during 2015–2018 are animated in S1 Movie. This animation shows that the 2015–2016 LSDV outbreaks tended to be restricted to the south of Russia, whereas starting from 2017 to 2018, outbreaks showed a substantial shift in the area of spread and steadily traveled eastward. The outbreaks started as a field-type epidemic and ended as a vaccine-like epidemic similar to outbreaks in 2018. In the animation, each dot on the map represents a LSDV outbreak: the blue dots indicate field isolates, the black dots undetermined, and the red dots vaccine-like isolates.

During 2015–2018, the outbreaks tended to peak in June–July and end in September–October. However, the 2018 outbreak extended till November (Fig 8).

## Discussion

In the present study, we provided an overview of LSDV evolution in Russia since the first incursions that began in North Caucasus in 2015 an extended Eastward along the border of Kazakhstan. In the current epidemiological scenario, the geographical spread of the outbreaks and the genetic profiles of the recovered isolates have changed.

Evolution of viruses drives genetic diversity and provides opportunities for species divergence [24] [37]. LSDV is a double-stranded DNA virus of the genus *Capripoxvirus*, which has exhibited relatively little genome evolution for up to 70 years [38]. However, viruses may follow a different evolutionary pathway upon coevolution with different replication-competent field and vaccine isolates [39] [40].

Here, the analysis of Russian LSDV isolates obtained during outbreaks in 2015–2018 is described. The first outbreaks were reported in North Caucasus in 2015 where no vaccination was practiced before (Fig 5). Resurgence of LSDV in 2016 [15] prompted the use of SSPV-based vaccines in Russia because these are considered safe, are devoid of virulence in cattle, and do not cause viremia. In this study, we showed that isolates from the affected regions in 2016 were identical to each other and were of the field isolate type (Figs 1–4). Furthermore, the isolate Dagestan/2015 [18] and 2016 isolates sequenced in the present study were identical based on selected gene targets both at the nucleotide and amino acid levels (Figs 1–4). The isolates Chechnya/2015, Stavropol/2016, Krasnodar/2016, Voronezh/2016, and Volgograd/2016 differed by a single G to T mutation in the *GPCR* locus, resulting in an amino acid change from cysteine to phenylalanine. The isolate Chechnya/2015 harbored an additional A to T nucleotide change, resulting in an amino acid change from serine to phenylalanine (Fig 1). Similar interchanges between two amino acids were shown to have a profound effect on mutated proteins [41]. Due to limited knowledge on LSDV proteins and their functions [1], it is challenging to predict the altered roles of *RPO30*. Isolates with such substitutions might have had a fitness advantage considering the geography (Chechnya, Stavropol, Krasnodar, Voronezh, and Volgograd are part of a shared area between North Caucasian and Southern parts of Russia) across which they spread (Fig 2); however, a particular mutation did not seem to be entrenched. With the introduction of vaccination with a Kenyan live vaccine in neighboring Kazakhstan in 2017, the first cases of disease from vaccine-like isolates were concurrently reported in the adjacent regions of Russia in 2017 onwards (Figs 6 and 7) [28].

Importantly, in 2018, no new field isolates (i.e., Dagestan/2015-like) were discovered (Figs 1–4). Instead, the pool of LSDV isolates was represented by vaccine-like isolates with divergent clustering patterns (Figs 1–4). As described in this study, since the *GPCR* locus of all isolates from 2018 carried the unique CTCAGTACAATT indel, they all tested positive for the vaccine strain in the assay developed by Agianniotaki et al. [42]. They were negative for a LSDV vaccine signature in LSDV008 using a PCR, which only detects commercial LSDV vaccine isolates [27].

Sequencing of the *RPO30* and *GPCR* loci, which detected incongruent grouping in Saratov/2017 [29], also indicated that Samara/1461/2018 and Kurgan/2018 (isolate not sequenced herein) were KSGP-like (Figs 1 and 2). However, the protein sequence of *RPO30* in Samara/1461/2018 was highly homologous to that of the field isolates, indicating a possible recombinant profile, similar to Saratov/2017 isolate (isolate not sequenced herein) (Fig 3). Detecting KSGP-like Samara/1461/2018 and Kurgan/2018 isolates (isolate not sequenced herein) actually circulating in the field sheds some light on the emergence of recombinant Saratov/2017, which indeed aligned with the Kenyan KSGP isolate rather than with Dagestan/2015 isolate [29, 18]. Further research is required to investigate the extent of contribution of KSGP-like isolates to the epidemiology of LSDV in Russia in 2018.

Omsk/2018, Omsk2/2018, Sverdlovsk/2018, and Chelyabinsk/2018 isolates exhibited different patterns of *RPO30* and *GPCR* target clustering from Saratov/2017 (Figs 1–4). However, Samara/1462/2018 clearly grouped with vaccine-like Samara/2017 isolate and the vaccine isolate Lumpyvax (commercial product) for both loci at both the nucleotide and amino acid levels (Figs 1–4); this pattern strongly suggests that Samara/2017 is a commercial vaccine isolate, although its mode of introduction into the country remains unknown and might have occurred through illegal vaccination. More importantly, such vaccine cases related to the identification of DNA belonging to viruses contained within commercial products such as Lumpyvax are extremely rare (Figs 1–4), rather the vaccine-like isolates reported in Russia in 2018 are of a recombinant nature. However, there is a lack of solid evidence to rule out the transmission of live Neethling vaccine viruses, and the steady Eastward movement of vaccine-like isolates in particular KSGP-like ones along the Russian border signals their transmission capacity.

Overall, the 2015–2018 outbreaks can be considered two distinct epidemics: a field-type epidemic in 2015–2016 and a vaccine-like epidemic in 2017–2018 (Figs 5–7). Genetically, although there were field isolates (Dagestan-like) [18] circulating in 2017 [28], they have been replaced by vaccine-like isolates carrying the genetic signatures of live commercial LSDV vaccine and KSGP-like isolates in 2018 (Figs 1 and 2). This genetic evidence clearly demonstrates that the 2017–2018 epidemic represents a novel emergence of LSDV in Russia rather than a continuation of the 2015–2016 epidemic (Figs 5–7).

The genetic diversity of LSDV in Russia observed over a period of a few years raises few important issues regarding the current virus evolution and vaccination strategies. It has been shown that DNA viruses, such as LSDV, are extremely conserved [38]. Moreover, vaccination with live attenuated vaccines has been conducted in several endemic countries, and no such cases of recombination have been detected to date for capripoxviruses. Although recombination in DNA viruses is not rare [43], with proper molecular tools and next-generation sequencing it becomes easier to determine recombination events and to trace the origin of outbreaks. Increasing amounts of available sequence data is expected to lead to a better understanding of genetic and biological traits of LSDV. However, since detection of a Neethling vaccine isolates is exempted from the World Organization for Animal Health notification [44], the actual situation of LSDV circulation worldwide remains heavily underrepresented.

The other key aspect of the complicated LSDV epidemiology is the origin of KSGP like isolates in Russia. Notably, the KSGP isolate has never been used in Russia, however the Kenyan Lumpivax for cattle has been used in neighboring Kazakhstan since 2017. The field circulation of KSGP like isolates has now been reported in Nigeria [45]. Sequencing of LSDV isolates recovered from outbreaks in 2018 demonstrated that LSDV variants indeed harbor KSGP-like sequences (Fig 1). The prevailing view that LSDV is only vector-borne [10] and the unavailability of the full-genome sequence of the Kenyan Lumpivax isolate greatly impede progress in ascertaining the contribution of this vaccine to the surge of vaccine-like isolates along the Russian border from 2017 onwards.

Overall, the vaccine-like isolates of LSDV have entrenched and eventually dominated the epidemiological situation in 2018, which is alarming. Indeed, the number of outbreaks associated with recombinant LSDV isolates along the Russian border is concerning since neighboring countries in these regions are currently officially free of LSDV, with vaccine-like isolates still prevalent along the border of Russia (Figs 6 and 7). In addition, LSDV outbreaks have not spread up North in Russia toward the gradient of cattle density. In contrast, they tend to travel along with a minimum distance of 1 km from the border (Figs 6 and 7).

Another important issue is the transmission capacity of live LSDV vaccines [24, 37]. Our literature search failed to identify studies on whether such vaccines are transmissible in the field. However, experience from the field survey reported here suggests that vaccine escape for LSDV does occur with negative consequences on the epidemiology of the disease [43]. Moreover, outbreaks in 2017 and 2018 were prominent in the warmer months (Fig 8), possibly corroborating the concept of vector-mediated spread of field and vaccine-like recombinant strains. However, the observed aggressive pattern cannot be attributed to *Stomoxys calcitrans* abundance because the fly appears mainly in autumn. The first generation of flies normally peaks in late July in Central Russia and Siberia [46, 47, 48], whereas LSD outbreaks frequently appear as early as May. Notably, in 2018, LSDV outbreaks were reported as late as November (Fig 8) in Urals and Siberia when there these flies are not active. For example, *Culicoides* midges–the most studied dipteran in Russia–do not appear beyond September due to the strong continental climate [49]. The observed identifications strongly indicate other transmission mechanisms, such as contact transmission or fomites, as a feasible explanation.

There is no doubt that approaches toward LSDV vaccination require an urgent overhaul worldwide in light of the findings reported here. Recombination among capripoxviruses, previously only a theoretical proposition, has recently been documented [29]. This report adds to the growing body of evidence that recombination in DNA viruses is readily possible and can generate hybrid progeny in the field causing disease [43]. Dominance of genetically divergent LSDV isolates compared with that of typical Dagestan/2015-like isolates in Russia in 2018 underscores certain favorable acquisitions in the genome of these novel isolates. An improved understanding of these viruses can be elucidated through whole-genome sequencing. Further studies are warranted to delineate when and where in the capripoxvirus genome such rearrangements may have occurred and what specific fitness advantages novel isolates have acquired related to transmission capacity. These data would lay the groundwork for future approaches to tunable attenuation through genetic engineering to develop safer vaccine preparations [50].

## Supporting information

S1 File (PDF)

S2 File (PDF)

S1 Movie (WEBM)

S2 Movie (WEBM)
