# Supplementary material for: Evidence of recombination of vaccine strains of lumpy skin disease virus with field strains, causing disease
Source: PLoS One. 2020 May 13;15(5):e0232584. doi: 10.1371/journal.pone.0232584 (PMC7219772; doi:10.1371/journal.pone.0232584)
Supplement: S1 File — (PDF) [file pone.0232584.s001.pdf]

Federal Service for Veterinary and Phytosanitary Surveillance
Federal State-Financed Institution
«Federal Centre for Animal Health»
(FGBI «ARRIAH»)
OIE Regional Reference Laboratory for Foot and Mouth Disease,
OIE Collaborating Centre for Diagnosis and Control of Animal Diseases
for the Countries of Eastern Europe, Central Asia and Transcaucasia,
FAO Reference Centre for FMD for Central Asia and West Eurasia

"10" January 2019

To PLOS one

With regard to the request concerning PONE-D-19-11170R2, we report the following:

1. The maps (Figures 5, 6 and 7) were built by the Information Ȧnalysis Centre under the Department for Veterinary Surveillance of FGBI ARRIAH (Vladimir, Russia) using the licensed ArcMap Desktop software version 10.7.1. For this purpose, shape files were retrieved from the open access web site (Natural Earth (http://www.naturalearthdata.com/).

2. The authors of the concerned manuscript have been given permission to use the corresponding figures built by the Information Analysis Centre (the proprietary logo is the top right corner). The figures have not been used in any previous publications.

The head of the
Information Analysis Centre

Karaulov A.K

600901 Yur'evets Vladimir Russia
Тел.: (4922) 26-06-14, Tel/Fax: (4922) 26-38-77
E-mail: mail@arriah.ru
